# Enhanced Skin Wound Healing Through Chemically Modified Messenger RNA Encoding Epidermal Growth Factor (EGF)

**DOI:** 10.1111/iwj.70143

**Published:** 2025-05-04

**Authors:** Haiyang Hu, Qianglong Sheng, Fan Yang, Xinyi Wu, Youlai Zhang, Shuling Wu, Yihu Liu, Ningyan Hu, Chenhong Fu, Jialin Leong, Rufei Deng, Zhenyu Jiang, Jiaxin Chen, Zhenxing Wang, Chunyuan Chen, Fei Chen, Yixuan Luo, Yuanlin Zeng, Yin Yu, Hui Xie, Gang Wang, Lijin Zou

**Affiliations:** ^1^ Jiangxi Provincial Key Laboratory of Trauma, Burn and Pain Medicine, Medical Center of Burn Plastic and Wound Repair, The First Affiliated Hospital, Jiangxi Medical College Nanchang University Nanchang Jiangxi China; ^2^ Department of Pharmacology Innovative Institute of Basic Medical Sciences of Zhejiang University Zhejiang Hangzhou China; ^3^ Geneheal Pharmacy Ltd Zhejiang Hangzhou China; ^4^ Nanchang University Queen Mary School Jiangxi China; ^5^ Department of Orthopedics, Movement System Injury and Repair Research Center, Xiangya Hospital Central South University Changsha Hunan China; ^6^ Hunan Key Laboratory of Angmedicine Changsha Hunan China; ^7^ National Clinical Research Center for Geriatric Disorders, Xiangya Hospital Central South University Changsha Hunan China; ^8^ Center for Materials Synthetic Biology, Shenzhen Institute of Synthetic Biology, Shenzhen Institute of Advanced Technology Chinese Academy of Sciences Shenzhen China; ^9^ CAS Key Laboratory of Quantitative Engineering Biology, Shenzhen Institute of Synthetic Biology, Shenzhen Institute of Advanced Technology Chinese Academy of Sciences Shenzhen China

**Keywords:** chemically modified mRNA, epidermal growth factor, protein enhancement therapy, wound healing

## Abstract

Efficient wound healing remains a formidable medical challenge in clinical practice, due to the prevalence of skin defects arising from diverse etiological factors. It is indisputable that epidermal growth factor (EGF) plays a pivotal role in wound repair. However, its clinical application through recombinant proteins encounters challenges, including a short half‐life in vivo and high production costs. Addressing these limitations, recent advancements in chemically modified mRNA (cmRNA) technologies offer a promising alternative. This study explores the utilisation of cmRNA in a biocompatible citrate‐saline formulation to encode EGF for therapeutic purposes, capitalising on the advantages of cmRNA's inherent stability and the formulation's compatibility with biological systems. CmRNA demonstrated high transfection efficiency in human immortalised keratinocyte (HaCaT) and normal human dermal fibroblasts (NHDF) cells (93.97% ± 1.25% and 90.37% ± 0.97%, respectively), resulting in efficient production of biologically active EGF protein. In vitro, EGF cmRNA significantly promoted HaCaT and NHDF cell cycle, proliferation and migration. In vivo, in vivo imaging system (IVIS) imaging of murine skin confirmed localised and sustained expression of Luciferase cmRNA, with signals detectable up to 11 days post‐injection. Immunohistochemistry revealed protein expression in both epidermal and dermal layers as early as 1 h post‐injection, peaking at 48 h, further corroborated by enzyme‐linked immunosorbent assay (ELISA). In a full‐thickness skin defect mouse model, EGF cmRNA significantly accelerated wound healing, with superior re‐epithelialisation observed compared to controls by Day 6. Mitogen‐activated protein kinase (MEK)/Extracellular signal‐regulated kinase (ERK) and Ki67 mRNA expression levels were markedly increased, both in vitro and in vivo. By Day 14, histological and immunohistochemical analyses revealed that EGF cmRNA outperformed recombinant human EGF (rhEGF), as indicated by enhanced formation of hair follicles and cutaneous glands, better‐organised collagen fibres, and a reduced collagen Type I/III ratio. No adverse effects were observed in major organs, confirming cmRNA's biosafety. These results highlight the therapeutic potential of EGF‐encoding cmRNA as an effective and safe alternative for enhancing wound healing.

**Summary**

- The study aimed to develop a novel therapeutic approach using chemically modified messenger RNA (mRNA) encoding epidermal growth factor (EGF) to enhance skin wound healing.
- A chemically modified mRNA encoding EGF was engineered and administered to evaluate its effectiveness in promoting wound healing through enhanced cell proliferation and migration.
- In vitro and in vivo experiments were conducted to assess the therapeutic impact of mRNA encoding EGF on wound healing, comparing it to conventional treatments.
- The study found that mRNA encoding EGF significantly accelerated wound closure, enhanced collagen deposition, and promoted the formation of skin appendages, suggesting its promising potential as an effective treatment for wound healing in the future.

## 1 Background

Skin defects, arising from diverse causal origins, often present a substantial challenge in clinical scenarios [1, 2]. Wound repair involves a complex interplay among various cells, cytokines, and growth factors, regulating critical processes like haemostasis, inflammation, cell migration, proliferation, and tissue remodelling [3, 4].

Epidermal growth factor (EGF), a pivotal growth factor, plays a crucial role in orchestrating wound healing processes. It not only promotes the proliferation, migration, and differentiation of repairing cells such as keratinocytes, fibroblasts, and vascular endothelial cells but also exerts influence on extracellular matrix components, thereby participating in the remodelling of damaged tissues to reduce scar formation [5, 6, 7, 8, 9]. Despite its scientifically proven ability to expedite wound closure, the clinical use of EGF via recombinant proteins is hampered by limitations including poor absorption, short in vivo half‐life, and high production costs.

To address these limitations, chemically modified mRNA (cmRNA) therapy has emerged as a novel gene therapy approach, gaining recognition for its transformative impact with the conferment of the Nobel Prize in Physiology or Medicine in 2023. Currently, various modification techniques are available to produce mRNA that is more stable, with lower immunogenicity and higher translation efficiency. These approaches generally include the following: optimising the open reading frame, 5′ and 3′ untranslated regions (UTR) sequences; utilising modified nucleosides and cap structures. The first two can significantly enhance the stability and translation efficiency of mRNA, while the latter can notably reduce its immunogenicity and further boost its stability and translation efficiency [10, 11]. In contrast to protein‐based therapy, mRNA‐based therapies avoid potential risks associated with exogenous protein application, such as allergies and inflammation, and enhance protein stability. Additionally, it can be mass‐produced via in vitro transcription (IVT) in cell‐free systems [12]. Moreover, unlike DNA‐based gene therapeutics, such as adeno‐associated virus (AAV)‐based therapy, which poses safety concerns due to potential genomic integration [13, 14, 15], mRNA‐based therapies offer versatility by circumventing nuclear entry, enabling precise tissue targeting and transient effects. This controllability renders mRNA‐based therapies more suitable for applications in tissue repair and regeneration [11, 12, 16, 17]. However, to our knowledge, there is currently no documented therapeutic use of cmRNA encoding EGF to promote wound healing.

**SCHEME 1:**
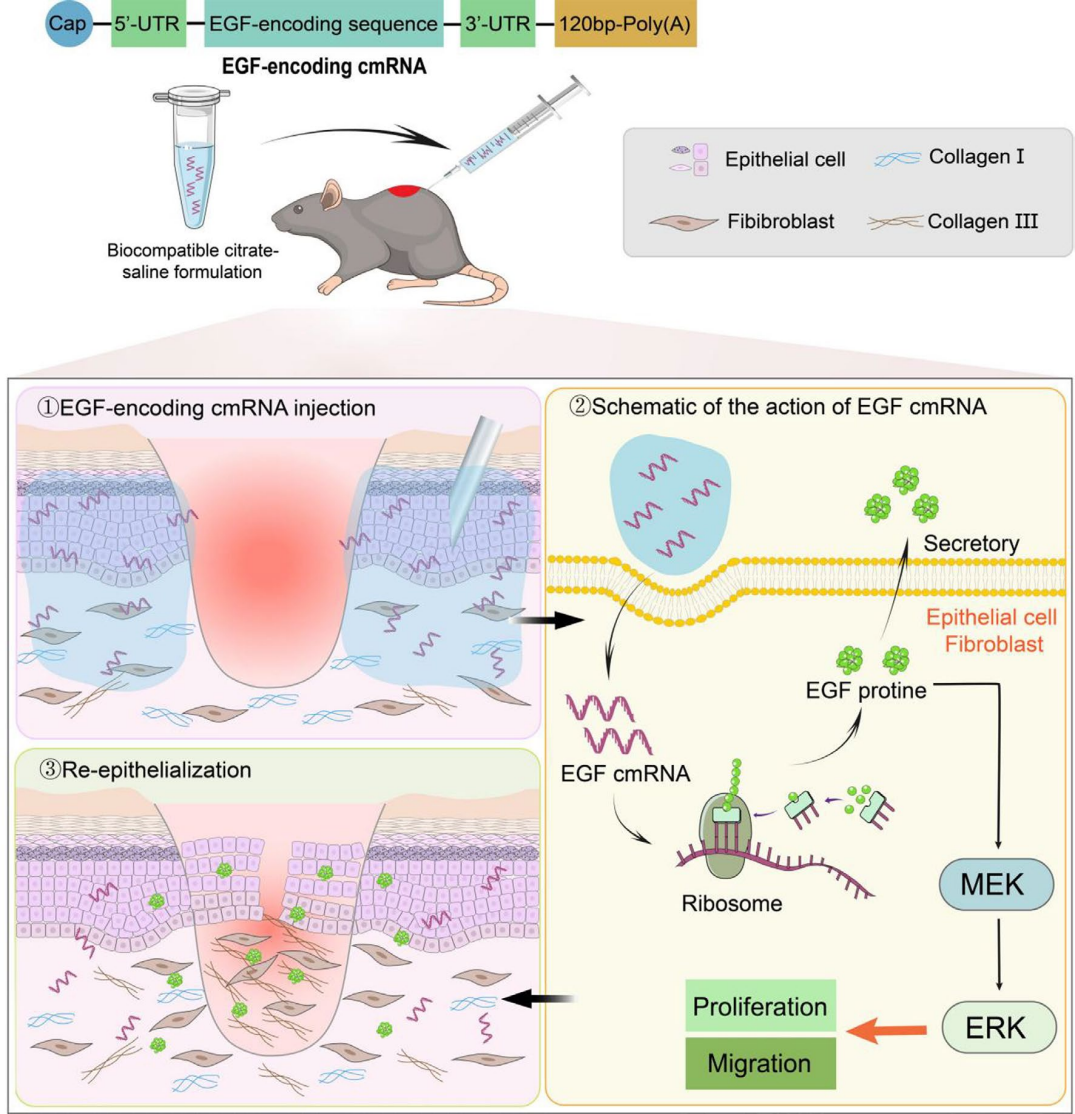
Illustration of how EGF‐encoded cmRNA in a biocompatible citrate buffer formulation functions in local tissue at the wound site.

The safe and effective delivery of mRNA to local tissue cells is a prerequisite for mRNA to exert its function. Multiple studies have indicated that a lipid‐based delivery system like lipid nanoparticles (LNPs), can result in infusion‐related hypersensitivity reactions, can be associated with tissue injury, and can promote RNA degradation via modulation of the local innate immune system [18, 19, 20, 21]. Recently, several teams utilised a cell‐based vascular endothelial growth factor (VEGF) cmRNA delivery platform to enhance therapeutic applications in limb ischemia, myocardial infarction, bone defects, and fat grafting [22, 23, 24, 25]. However, cell therapy still faces numerous limitations, including issues with cell sourcing, immune reactions, safety, and ethical concerns and so on, which hinder its large‐scale clinical application [26, 27, 28]. While Luo et al. utilised small skin as a carrier for stromal cell‐derived factor 1 alpha (SDF1α) cmRNA application in wound healing, achieving certain efficacy, but also encountered the aforementioned challenges [29]. The citrate buffer has been validated in different animal models and tissues in good biocompatibility [10]. In this report, we utilise citrate buffer and naked EGF‐encoding cmRNA formulation to resolve skin defects and accelerate wound healing, (Scheme 1).

## 2 Materials and Methods

### 2.1 In Vitro Transcription of cmRNA

As per prior methodologies [30], the amalgamation of the 5′ untranslated region (UTR), open reading frame, and 3′ UTR sequences into an empty vector initiated the process. Subsequently, *Escherichia coli* ‐mediated amplification of the plasmid occurred. Post‐amplification, restriction enzyme digestion ensued, and a poly‐(A) tail was affixed using PCR methodology employing Tail PCR primers (Table S1). The T7 co‐transcription RNA synthesis kit (10 111, Synthgene, Jiangsu, China) facilitated the in vitro transcription of the tailed PCR products. During transcription, cytidine and uridine underwent substitutions with 5‐methylcytidine and N1‐Me‐Pseudouridine, respectively. Cap1 mRNA capping was employed. Subsequent to transcription, DNase treatment eliminated residual DNA templates, followed by dephosphorylation via Antarctic Phosphatase (New England Biolabs). Purification utilising lithium chloride (S125‐01A, Yeasen, Shanghai, China) was conducted. Dilution to the requisite concentration transpired in a biologically compatible citrate‐saline buffer (10 mmol/L citrate, 130 mmol/L sodium chloride in RNase‐free water, pH adjusted to approximately 7.5 with sodium hydroxide), validated for biocompatibility across diverse animal models and tissues [10]. Quantification and assessment of mRNA quality occurred using NanoDrop, complemented by purity and integrity verification via agarose gel electrophoresis. The reporter gene enhanced green fluorescent protein (eGFP) and luciferase (Luc) cmRNA sequences adhered to established protocols [31]. The open reading frame sequence for EGF‐encoding cmRNA is available in Table S2, while the UTR sequence is detailed in Table S3.

### 2.2 Cell Isolation and Culture

The HaCaT cell line (Cobioer No.: CBP60331) was obtained from Cobioer Biosciences Co. Ltd., Nanjing, China. NHDF sourced from discarded skin specimens, collected from skin flap surgery of patients who provided informed consent after obtaining ethical approval, underwent isolation per literature guidelines [32]. Segmentation of skin tissue into smaller fragments initiated the culture process in Dulbecco's Modified Eagle's Medium (DMEM) supplemented with 10% fetal bovine serum (FBS) and 1% penicillin–streptomycin antibiotics. Cultivation ensued in a controlled environment at 37°C with periodic medium changes every 3 days. Passage of cells occurred upon reaching 90% confluence, utilising passages fewer than five for all experiments.

### 2.3 In Vitro Transfection Assay

Before transfection, cells were seeded at a density of 2 × 10^5^ cells per well in a six‐well plate and incubated overnight. On the following day, the cell culture medium was replaced with optimised minimum essential medium (OPTI‐MEM) (Gibco, Life Technologies, USA). Subsequently, with reference to the instruction manual, 5 μL of Lipofectamine MessengerMAX reagent (Invitrogen, Life Technologies, USA) was mixed with 95 μL of OPTI‐MEM. The mixture was left at room temperature for 5 min. Additionally, for the Luc and EGF cmRNA groups, 2 μL of mRNA (1 μg/μL) was mixed with 98 μL of OPTI‐MEM, and for the NC group, 100 μL of OPTI‐MEM without any cmRNA. The Lipofectamine MessengerMAX dilution and the RNA dilution were thoroughly mixed to prepare an RNA‐lipid suspension, which was incubated at room temperature for 15 min. The RNA‐lipid suspension was then evenly added to the cells. After 4 h, the OPTI‐MEM was replaced with complete growth medium. For the assessment of transfection efficiency, 24 h post‐transfection, cells were detached using 0.25% trypsin, collected, washed twice with phosphate‐buffered saline (PBS), and centrifuged. Cell density was adjusted to 5 × 10^5^/mL, and 500 μL of cell suspension was placed in a 2 mL centrifuge tube. Flow cytometry was utilised to detect the transfection efficiency of eGFP in the cells.

### 2.4 Enzyme‐Linked Immunosorbent Assay

The investigation into the kinetics of EGF‐encoding cmRNA expression was facilitated by using an EGF Elisa kit (KHG0061, Invitrogen, Life Technologies, USA). In vitro, following transfection of HaCaT and NHDF cells, a time‐dependent collection of cell culture supernatants was performed at specific intervals (6, 12, 24, 36, 48, 72, 96, 120 h). Subsequently, quantification of EGF protein levels was carried out via enzyme‐linked immunosorbent assay (ELISA) analysis. For in vivo assessments, an intradermal injection of 25 μg of EGF‐encoding cmRNA (2.5 μg/μL) into the dorsal skin of mice was administered. Post‐injection, skin tissue samples were obtained from the injection site for total protein extraction using a skin tissue protein extraction kit (SA‐01‐SK, Invent Biotechnologies, Beijing, China), followed by ELISA analysis to quantify EGF protein levels.

### 2.5 Proliferation Assay

Evaluation of the influence of EGF‐encoding cmRNA on skin cell proliferation was conducted using the cell proliferation assay with the Cell Counting Kit‐8 (CCK‐8 Kit) (C0042, Beyotime, Shanghai, China). Cells in the logarithmic growth phase were seeded into 96‐well plates, with HaCaT cells at a density of 3000 cells per well and NHDF cells at 1000 cells per well (100 μL/well). Following a 24‐h incubation, cells were transferred to OPTI‐MEM medium before separate transfections with Luc and EGF‐encoding cmRNA. After a 4‐h transfection period, a transition to a low serum culture medium containing 1% FBS occurred. The addition of CCK‐8 reagent (10 μL) at 2 and 4 days post‐transfection was followed by a 2‐h incubation period, and absorbance was measured at 450 nm using an enzyme‐linked immunosorbent assay reader.

### 2.6 Cell Cycle Assay

The impact of EGF‐encoding cmRNA on the cell cycle of epidermal and fibroblast cells was assessed through flow cytometry analysis. Cells seeded in six‐well plates underwent cmRNA transfection as per established protocols. Following a 48‐h post‐transfection interval, cells were harvested, washed with pre‐cooled PBS, and fixed in pre‐cooled 70% ethanol for 2 h. Subsequent PBS washing was followed by incubation with RNase (10 mg/mL) (10407ES60, Yeasen, Shanghai, China) and propidium iodide (PI) (5 mg/mL) (ST511, Beyotime, Shanghai, China) under light‐restricted conditions. Incubation in the dark at room temperature for 30 min preceded flow cytometry analysis to detect red fluorescence at an excitation wavelength of 488 nm. Data analysis was conducted using FlowJo software v10.8.1.

### 2.7 Wound Healing Assay In Vitro

A wound‐healing experiment was conducted to evaluate the influence of transfected EGF‐encoding cmRNA on wound closure in vitro. Cells were cultured in six‐well plates, with HaCaT cells seeded at a density of 1 × 10^6^ cells per well and NHDF cells at 4 × 10^5^ cells per well. Upon reaching approximately 100% confluence on a subsequent day, a scratch was induced using a 100 μL pipette tip, and the medium was replaced with a low serum culture medium containing 1% FBS. HaCaT cell images were captured at 0, 12, 18, and 24 h post‐scratch, while NHDF cell images were taken at 0, 12, 24, and 48 h post‐scratch. Image J software (NIH, Bethesda, MD, USA) facilitated wound area measurements. The wound healing rate (%) was computed using the formula: Wound healing rate% = (Initial area—Remaining area)/Initial area × 100%.

### 2.8 Bioluminescence Imaging Studies

Utilising IVIS Spectrum Imaging (PerkinElmer, USA), bioluminescent imaging was conducted to assess cmRNA expression duration and in vivo distribution post intradermal injection. Male C57BL/6 mice (*n* = 3 per group, 8–10 weeks old) were anaesthetised using isoflurane (R510, RWD, Shenzhen, China), and dorsal fur was removed. Subsequent intradermal injections of 0, 7.5, and 25 μg Luc cmRNA were administered. Six hours post‐injection, mice were intraperitoneally injected with D‐luciferin sodium solution (Cat No. 40901ES01; Yeasen, Shanghai, China) at a dose of 150 mg/kg. Bioluminescent imaging using the IVIS system occurred 10 min post‐D‐luciferin administration. Repeated imaging took place daily until no fluorescent signal was detected. Image analysis utilised Living Image software (PerkinElmer).

### 2.9 Wound Healing Assay In Vivo

All animal experiments were executed in accordance with the approved protocols by the Institutional Animal Care and Use Committee of the First Affiliated Hospital of Nanchang University, ensuring humane treatment and adherence to ethical standards. The study aimed to explore the in vivo wound healing effects of EGF‐encoding cmRNA using a full‐thickness skin defect model in mice. Fifty 6–8‐week‐old male C57BL/6 mice were obtained from Hangzhou qizhen Laboratory Animal Technology Co. Ltd. Following anaesthesia with 1% pentobarbital sodium, dorsal hair removal was performed, ensuring complete hair elimination using a depilatory cream. Subsequent disinfection with iodine preceded the creation of a full‐thickness skin defect using an 8 mm diameter skin punch. Injections of either mRNA solution (0.75 or 2.5 mg/mL in 10 mmol/L citrate, 130 mmol/L saline) or citrate‐saline vehicle (10/130 mmol/L) were administered around the wound perimeter on Days 0 and 3 at various angles as shown in Figure S1. For the recombinant protein group, 50 μL of recombinant EGF protein (1 μg/μL in 0.9% saline with 0.1% bovine serum albumin; R&D Systems) solution was administered directly to the wound surface, and the dose was repeated at 0, 2, 4, 6, 8, 10, and 12 days after surgery. Wounds were photographed, covered with sterile Tegaderm (3M) dressings, and bandaged. Subsequent photographs taken on specified days were analysed using Image J software.

On Days 6 and 14, five mice from each group were randomly selected and euthanised. Wound tissues were assessed using Haematoxylin and Eosin (H&E) staining and qPCR for MEK/ERK and Ki67 expression on Day 6, and H&E, Masson staining, and Immunohistochemistry on Day 14. A flow chart demonstrating the animal study design is provided in Figure S2.

### 2.10 Histology Analysis

Wound tissue specimens were fixed in 4% paraformaldehyde at room temperature for 48 h, followed by triple washing in PBS. Subsequent tissue dehydration was accomplished utilising an ASP200S automatic dehydrator. Embedding of tissues in paraffin was performed using the BMJ‐1 tissue embedding system, followed by sectioning to 5 μm thickness employing the Leitz 1516 instrument. Post‐sectioning, slides underwent dewaxing, rehydration at 65°C, and preparation for staining. H&E staining involved sequential immersion of sections in haematoxylin solution for 300 s, differentiation solution for 180 s, and distilled water rinse. Following this, eosin staining solution was applied for 60 s. Subsequent dehydration was conducted before sealing with a neutral resin. Masson's trichrome staining employed the Masson staining kit (G1006, Servicebio, Wuhan, China). Post‐staining, standard dehydration steps were undertaken, followed by sealing with a neutral resin. Image capture of stained sections was facilitated using a microscope equipped with a DP71 camera (model BX51, Olympus, Japan). Measurements of epidermal thickness, collagen density, and cutaneous glands and hair follicles in the high‐power field (HPF) were performed with Image J software (National Institutes of Health, Bethesda, MD, USA) [33].

### 2.11 Immunohistochemistry

Following dewaxing and rehydration, antigen retrieval was performed employing high‐temperature and high‐pressure treatment. Endogenous peroxidase activity was quenched using a 3% hydrogen peroxide solution. Nonspecific binding blockade was executed with 10% goat serum albumin for 30 min, succeeded by PBS washes. Sections were then incubated overnight at 4°C with primary antibodies: mouse anti‐EGF antibody (1:100, sc‐166 779, Santa Cruz Biotechnology, Dallas, USA), rabbit anti‐type I collagen antibody (1:200, GB11022, Servicebio, Wuhan, China), and rabbit anti‐type III collagen antibody (1:200, GB111629, Servicebio, Wuhan, China). The subsequent day involved PBS washes to remove excess primary antibodies, followed by application of secondary antibodies: goat anti‐rabbit IgG H&L (HRP) (1:400, G1213, Servicebio, Wuhan, China) and goat anti‐mouse IgG H&L (HRP) (1:400, GB23301, Servicebio, Wuhan, China). Chromogenic detection was accomplished using freshly prepared DAB coloration solution (G1212, Servicebio, Wuhan, China) and counterstaining with haematoxylin. Following dehydration, sections were clarified and sealed using a neutral resin prior to imaging using a microscope equipped with a DP71 camera (BX51, Olympus, Tokyo, Japan). The integrated optical density (IOD) was measured using the Image J software to indicate the protein expression levels.

### 2.12 Quantitative Reverse Transcription Polymerase Chain Reaction

Total RNA isolation from cells and tissues utilised the Total RNA Kit (19221ES50, Yeasen, Shanghai, China), followed by cDNA synthesis using the reverse transcription kit (R312, Vazyme, Nanjin, China). The quantitative reverse transcription polymerase chain reaction (qRT‐PCR) cycles involved an initial denaturation step at 95°C for 600 s, followed by 40 cycles at 95°C for 15 s and 60°C for 60 s on the Bio Rad real‐time PCR system (Bio Rad, CA, USA). Normalisation of Ct values to GAPDH and computation of relative gene expression levels employed the 2^−ΔΔCt^ method. Primer details for human and mouse MEK, ERK, and Ki67 genes are provided in Tables S4 and S5.

### 2.13 Statistical Analysis

For comparing differences between two independent groups, a two‐tailed *t*‐test was employed. In cases involving comparisons across multiple sample groups, one‐way analysis of variance (ANOVA) was utilised to identify significant intergroup differences. Experimental data were presented as mean ± standard deviation (SD) and statistical analyses and graphing were performed using GraphPad Prism 9 software (La Jolla, CA, USA). *p* Values < 0.05 were considered statistically significant. Each experiment was conducted at least three times independently to ensure the reliability of the results.

## 3 Results

### 3.1 Efficiency and Protein Kinetics of cmRNA Transfection in HaCaT and NHDF

**FIGURE 1:**
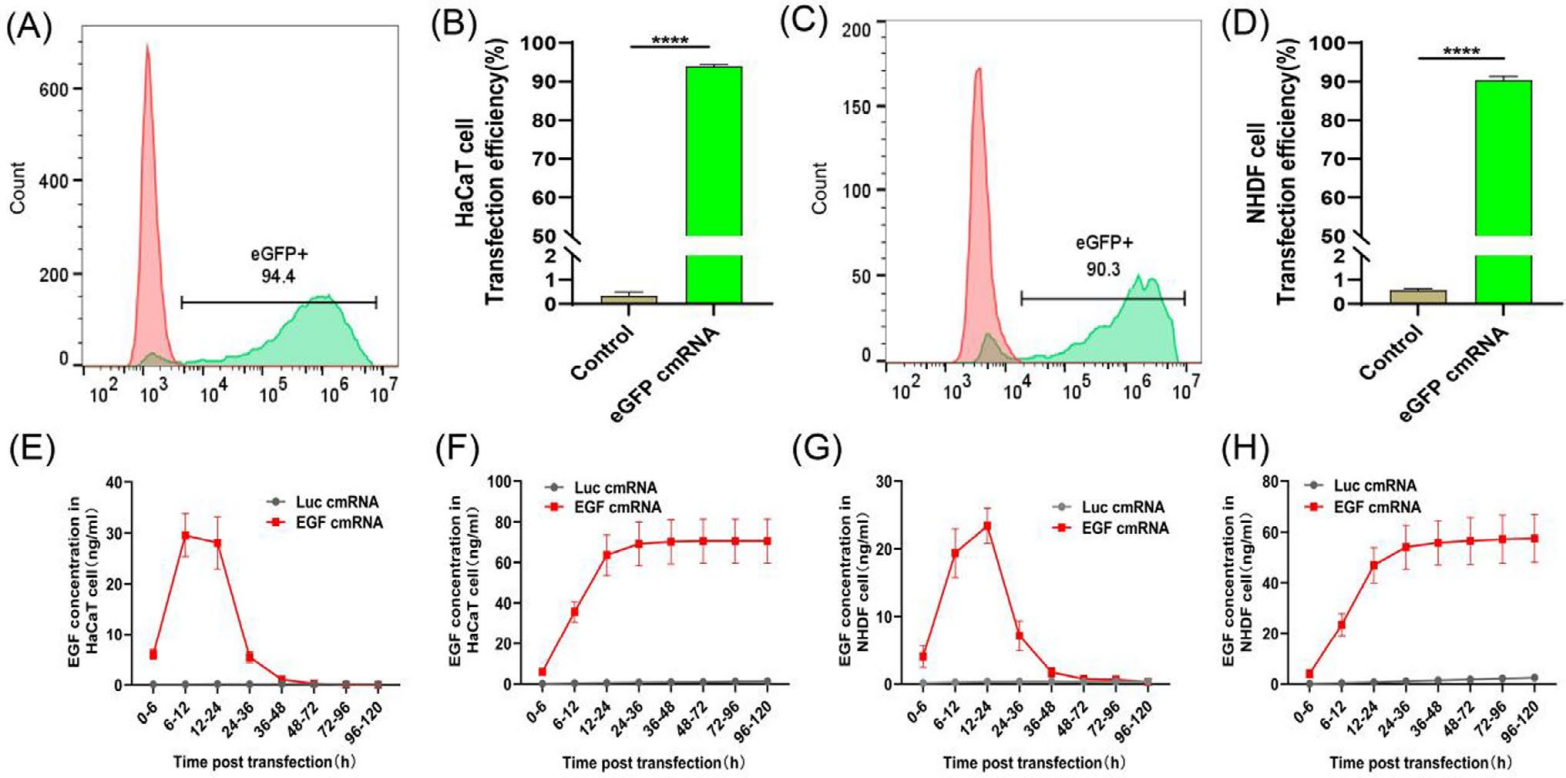
The transfection efficacy and temporal kinetics of protein expression subsequent to cmRNA transfection in HaCaT and NHDF cellular models. (A) and (B) Flow cytometric analysis of transfection efficiency of eGFP‐encoding cmRNA in HaCaT cells. (C) and (D) Flow cytometric analysis of transfection efficiency of eGFP‐encoding cmRNA in NHDF cells. (E) and (F) Time course of EGF protein expression and accumulation from HaCaT transfected with EGF‐encoding cmRNA. (G) and (H) Time course of EGF protein expression and accumulation from NHDF transfected with EGF‐encoding cmRNA. Data presented as mean ± SD (*n* = 5). Statistical significance is indicated as (****p < 0.0001).

First, we transfected eGFP cmRNA into both epidermal and fibroblast cells, and assessed the transfection efficiency using flow cytometry. Our data demonstrate that eGFP cmRNA efficiently transfects epidermal cells (93.97% ± 1.25%) and fibroblasts (90.37% ± 0.97%) (Figure 1A–D). To analyse the protein expression dynamics of EGF‐encoding cmRNA in both epidermal cells and fibroblasts, we collected cell culture supernatants at different time points and used ELISA to measure the levels of EGF protein expression. Our results indicate that EGF‐encoding cmRNA achieves effective expression in both cell types. In HaCaT cells, EGF protein expression peaked within 6–12 h, whereas in NHDF cells, EGF protein expression reached its peak within 12–24 h (Figure 1E–H).

### 3.2 The Effect of EGF‐Encoding cmRNA on Cell Proliferation and Migration In Vitro

**FIGURE 2:**
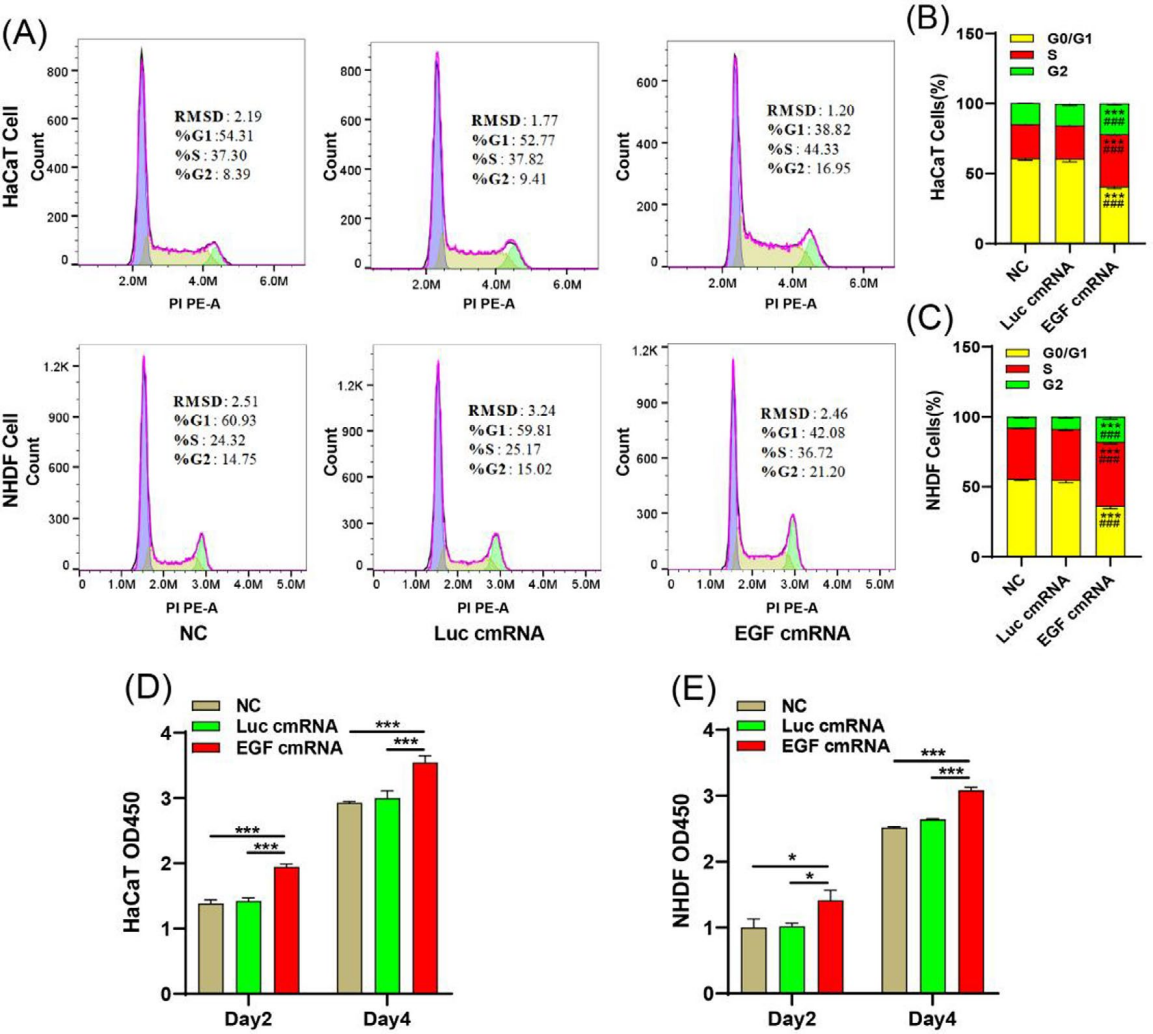
The impact of EGF‐encoding RNA on the proliferative behaviour of HaCaT and NHDF cells. (A) The influence of EGF‐encoding cmRNA on the cell cycle dynamics of HaCaT and NHDF cells. (B) and (C) Quantification of the percentage of cell numbers in each cycle at 48 h after transfection of HaCaT and NHDF cells. (D) and (E) The effects of EGF‐encoding cmRNA on the proliferation of HaCaT and NHDF cells at different time by CCK‐8 method. Data are presented as the mean ± standard deviation (*n* = 3), with statistical significance denoted by * (*p* < 0.05), *** (*p* < 0.0001) compared to the negative control (NC), and ### (*p* < 0.001) compared to Luc‐encoding cmRNA.

To evaluate the influence of EGF‐encoding cmRNA on cell proliferation, we initially examined the cell cycle 2 days after transfection using flow cytometry. The results indicated a significant increase in the number of cells in the S and G2 phases in both cell types following EGF‐encoding cmRNA transfection compared to the NC and Luc groups (Figure 2A–D). To more directly observe the influence of EGF on cell quantity growth, we employed the CCK‐8 assay to measure cell quantities of both cell types 2 and 4 days after transfection. The absorbance at 450 nm was directly proportional to the cell quantity. The data demonstrated a gradual increase in cell numbers with prolonged culture time. Notably, in both cell types, the EGF‐encoding cmRNA group exhibited a substantial increase in cell quantity compared to the NC and Luc cmRNA groups, with significant differences observed 2 and 4 days after cell transfection (Figure 2E,F). These findings collectively indicate that EGF‐encoding cmRNA effectively promotes the proliferation of both epidermal and fibroblast cells.

**FIGURE 3:**
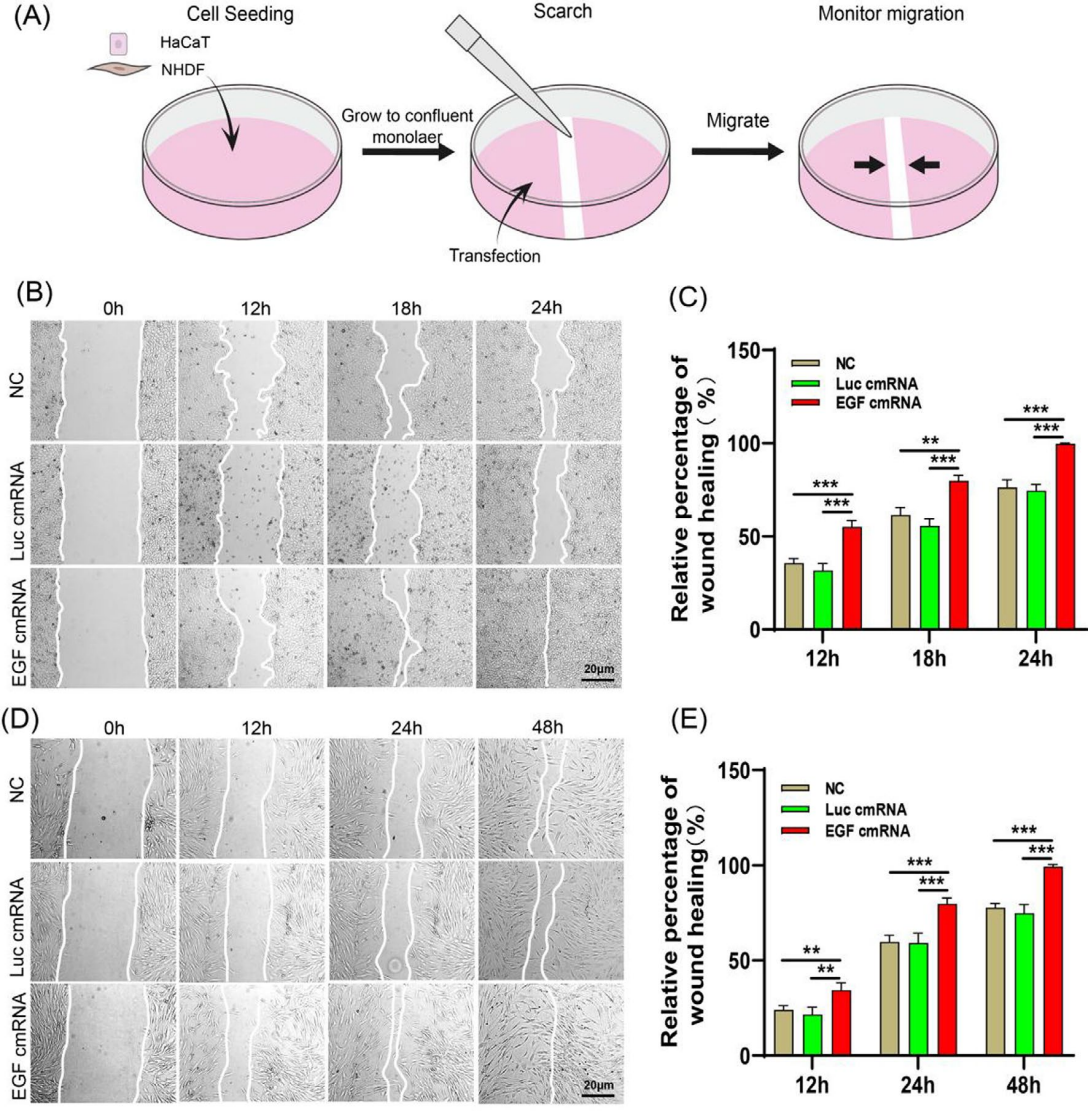
The impact of epidermal growth factor chemically modified RNA (EGF‐encoding cmRNA) on in vitro wound healing is illustrated through representative images and quantitative analysis. (A) Experimental diagram for wound healing in vitro. (B) and (C) Representative images and quantitative analysis of HaCaT migration after treatment with EGF‐encoding cmRNA. (D) and (E) Representative images and quantitative analysis of NHDF migration after treatment with EGF‐encoding cmRNA. Data presented as mean ± SD (*n* = 6). Statistical significance is indicated as (**p < 0.01 and ***p < 0.001).

To evaluate the influence of EGF‐encoding cmRNA on wound healing in vitro, we employed a cell scratch assay to simulate wound healing and evaluate the impact of EGF‐encoding cmRNA transfection on cell migration(Figure 3A). As our results demonstrate, EGF‐encoding cmRNA significantly enhances the migratory capabilities of both epidermal and fibroblast cells. For epidermal cells, we observed wound closure at 12, 18 and 24 h post‐transfection. The results showed that the EGF‐encoding cmRNA group exhibited a significantly higher wound closure rate at all time points compared to the NC and Luc groups. Notably, the EGF‐encoding cmRNA group achieved near‐complete wound closure at 24 h, while the other two groups had not fully closed(Figure 3B,C). In contrast, fibroblast cell migration was relatively slower. Thus, we observed wound closure at 12, 24 and 48 h post‐transfection. Similar to epidermal cells, the EGF‐encoding cmRNA group demonstrated significantly higher wound closure rates at all observation time points compared to the NC and Luc groups. At 48 h, the EGF‐encoding cmRNA group had also achieved near‐complete wound closure, while the NC and Luc groups had not fully closed (Figure 3D,E). These findings indicate that EGF‐encoding cmRNA transfection significantly accelerates wound healing in vitro.

In the intricate journey of wound healing, the intrinsic ability of cells to both proliferate and migrate efficiently to the wound site stands as a cornerstone for successful tissue regeneration and repair [3, 4]. This cellular dynamism ensures that the wound is rapidly closed, minimising the risk of infections and other complications. The HaCaT and NHDF cells, representing epidermal and dermal cell types respectively, play pivotal roles in this regenerative process. Our findings, which highlight a significant enhancement in the proliferation and migration capacities of these cells post‐transfection with EGF cmRNA, underscore the potential therapeutic implications of this approach.

### 3.3 The Kinetics and Distribution of cmRNA Expression In Vivo

**FIGURE 4:**
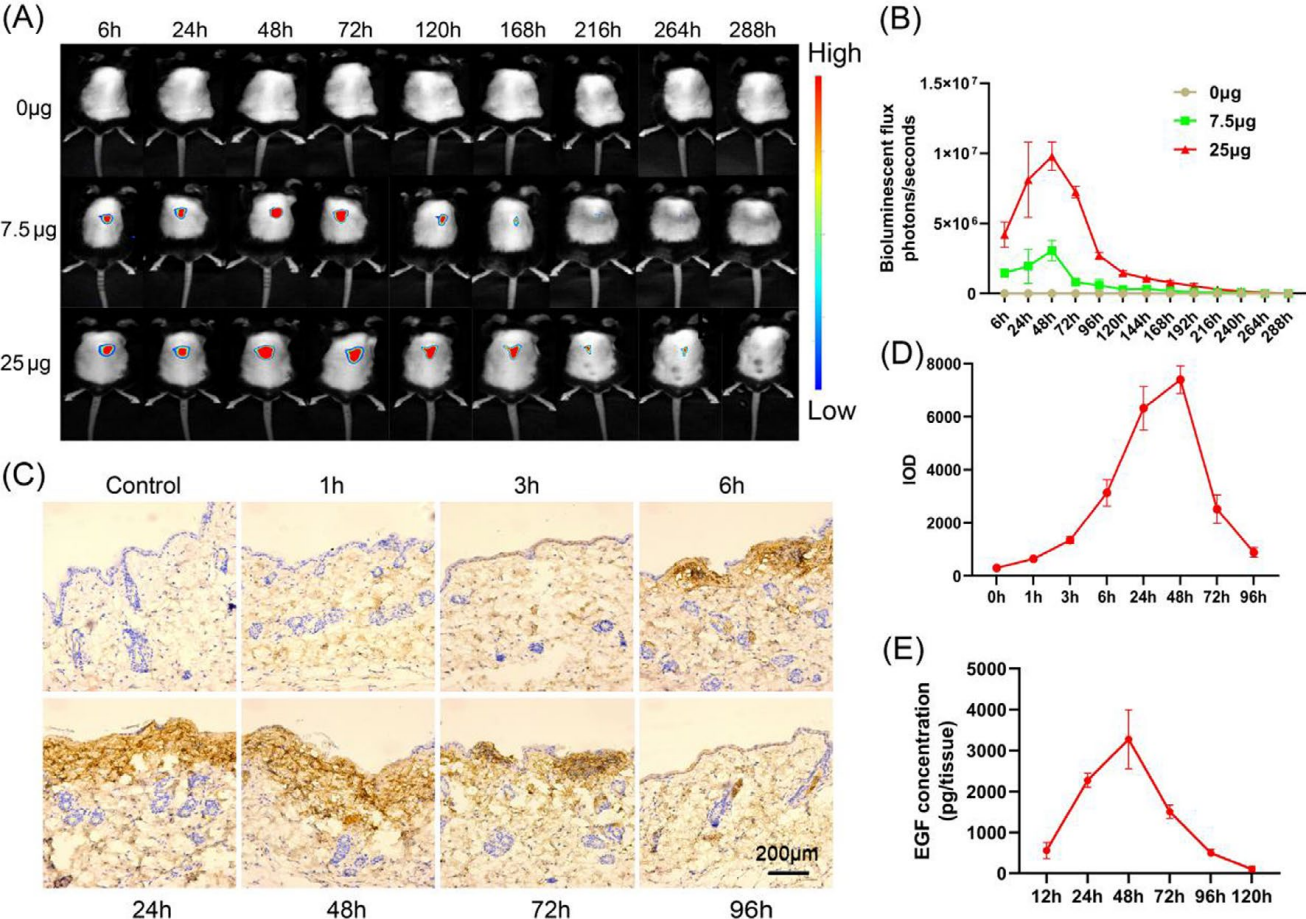
The kinetics and distribution of cmRNA expression in vivo. (A) Intradermal injections of 7.5 and 25 μg Luc cmRNA into the dermal layer, with representative images captured at various time intervals spanning 11 days. (B) Semi‐quantitative analysis of the fluorescence intensity (*n* = 3) observed from the injected Luc cmRNA. (C) Representative images obtained from immunohistochemistry conducted at different time points within a 96‐h timeframe. (D) Quantification of the immunohistochemical data. (E) Pharmacokinetics of protein expression following a single intradermal injection of Epidermal Growth Factor (EGF) cmRNA over 120 h in mice (*n* = 3 per time point). All data are presented as the mean ± standard deviation (*n* = 3).

We next prepared cmRNA in citrate buffer and administered it intradermally into mouse skin tissue. Utilising a small animal imaging system, we monitored the in vivo expression kinetics of cmRNA. Our results indicate that Luc cmRNA can achieve local, long‐lasting expression in the skin, peaking at 48 h and lasting up to 11 days (Figure 4A,B). Importantly, expression was not detected in other organ tissues, highlighting the safety of cmRNA intradermal injection.

To observe the distribution of EGF‐encoding cmRNA expression within skin tissue, we employed immunohistochemistry to assess expression at different time points following intradermal injection. Our data demonstrate that EGF‐encoding cmRNA exhibits minimal expression within 1 h post‐injection, reaches a peak at 48 h, and gradually decreases thereafter (Figure 4C,D). This trend is consistent with the small animal imaging results and is corroborated by ELISA data (Figure 4E). Besides, we observed widespread distribution of the expressed proteins throughout the entire skin layer, which is not attainable via recombinant protein applications.

### 3.4 The Effect of EGF‐Encoding cmRNA on Wound Healing In Vivo

**FIGURE 5:**
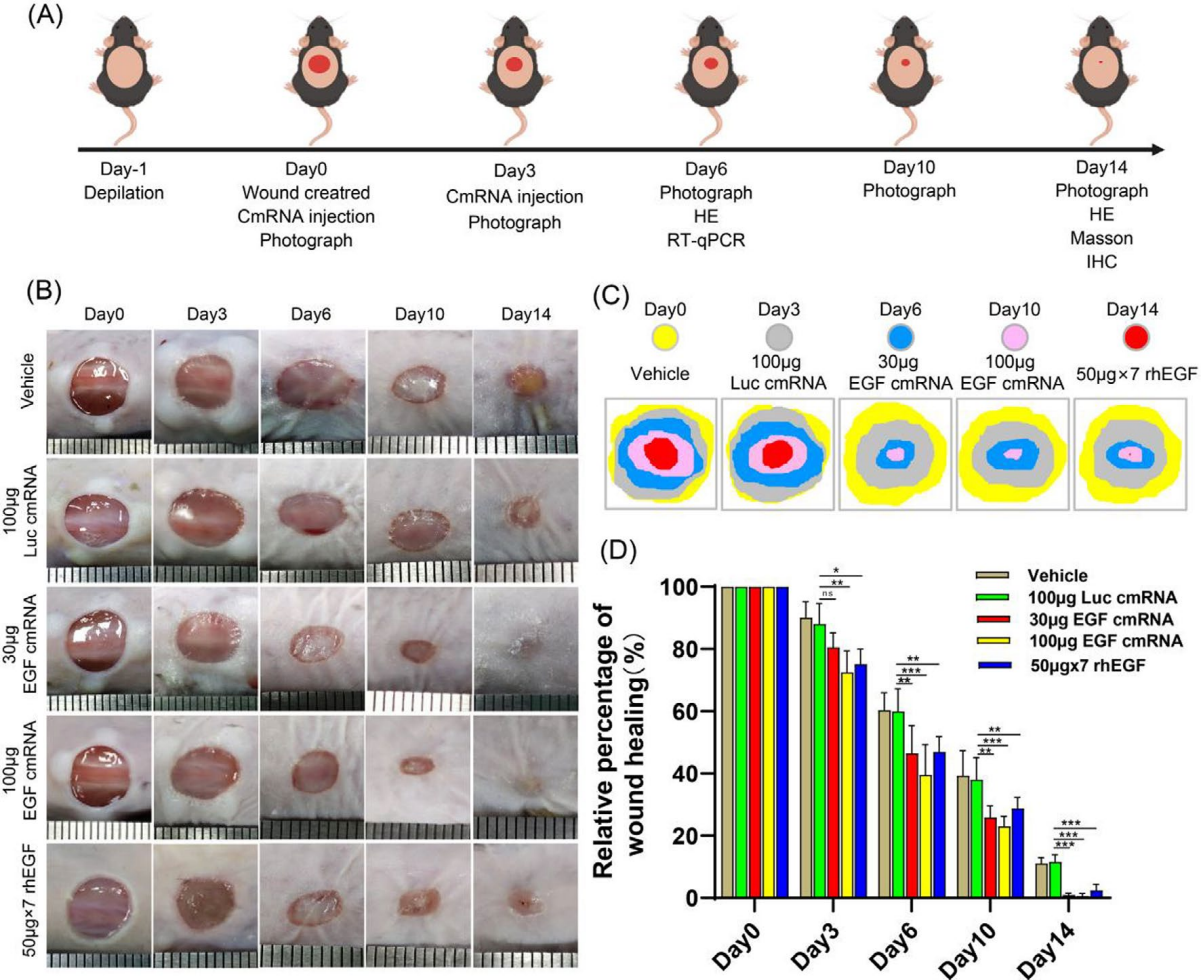
The effect of EGF‐encoding cmRNA on wound healing in vivo. (A) Depiction of a schematic illustrating the sequence of animal experiments conducted to evaluate the therapeutic efficacy of EGF‐encoding cmRNA for skin wound healing. (B) Depiction of representative images portraying wound progression at distinct time intervals (*n* = 5). (C) A corresponding schematic illustrating changes in wound area following various treatments on Days 3, 6, 10, and 14. (D) Relative quantification of wound healing percentages (%) across different time points post‐treatment. Data presented as mean ± SD (*n* = 5). Statistical significance is indicated as (*p < 0.05, **p < 0.01 and ***p < 0.001).

Based on the previously established biological activity of EGF‐encoding cmRNA in in vitro experiments and the reliability of its expression in skin tissue in vivo, we have reason to believe that EGF‐encoding cmRNA can also promote the proliferation and migration of epidermal and fibroblast cells in vivo, thereby accelerating wound healing. To investigate this hypothesis, we constructed a full‐thickness skin defect model in mice with an 8 mm diameter. Citrate buffer (Vehicle) and Luc cmRNA were used as negative controls, rhEGF was used as a positive control, and due to its short half‐life in vivo, it was administered at a dose of 50 μg every other day [34]. In contrast, cmRNA has a longer expression duration, and based on our data above and findings described in [35], we chose to administer it twice at doses of 30 and 100 μg. The therapeutic approach is depicted in Figure 5A. In injections were given at different locations on Days 0 and 3 (Figure S2). Representative images of the wounds on Days 0, 3, 6, 10, and 14 are shown in Figure 5B. Moreover, a schematic diagram of the wound area over time are shown in Figure 5C. As illustrated in Figure 5B, it can be observed that the wound healing rate for the EGF‐encoding cmRNA and the recombination human EGF (rhEGF) treatment groups is noticeably accelerated. By the 3rd day, a significant contraction is evident in all EGF treatment groups. After 14 days of treatment, all groups exhibited a reduction in wound size. Wounds in all EGF treatment groups have fully epithelialised, while the vehicle and Luc groups still had open wounds.

By calculating the healing rates at each time point (Figure 5D), it was observed that higher doses of EGF‐encoding cmRNA accelerated early wound healing. On Day 3 post‐surgery, the 100 μg EGF‐encoding cmRNA group (72.6% ± 6.8%) and the rhEGF group (75.1% ± 4.8%) exhibited significantly smaller open‐wound sizes compared to the vehicle group (90.1% ± 5.1%) and the Luc cmRNA group (88.0% ± 6.7%). On Day 6, the 30 μg EGF‐encoding cmRNA group (50.8% ± 4.0%), the 100 μg EGF‐encoding cmRNA group (47.1% ± 4.0%), and the rhEGF group (50.7% ± 3.8%) all displayed significantly smaller open‐wound dimensions. Likewise, on Day 10, the 30 μg EGF‐encoding cmRNA group (26.0% ± 3.8%), the 100 μg EGF‐encoding cmRNA group (23.1% ± 3.2%), and the rhEGF group (28.8% ± 3.5%) exhibited open wounds that were notably smaller than those of the vehicle group (39.3% ± 8.0%) and the Luc cmRNA group (38.0% ± 7.1%). By Day 14, the 30 μg EGF‐encoding cmRNA group (1.1% ± 0.0%), the 100 μg EGF‐encoding cmRNA group (0% ± 0.0%), and the rhEGF group (2.5% ± 0.5%) all demonstrated significantly increased wound closure compared to the vehicle group (11.1% ± 1.8%) and the Luc cmRNA group (11.6% ± 2.3%).

### 3.5 Histological Analysis of Wound Tissues

**FIGURE 6:**
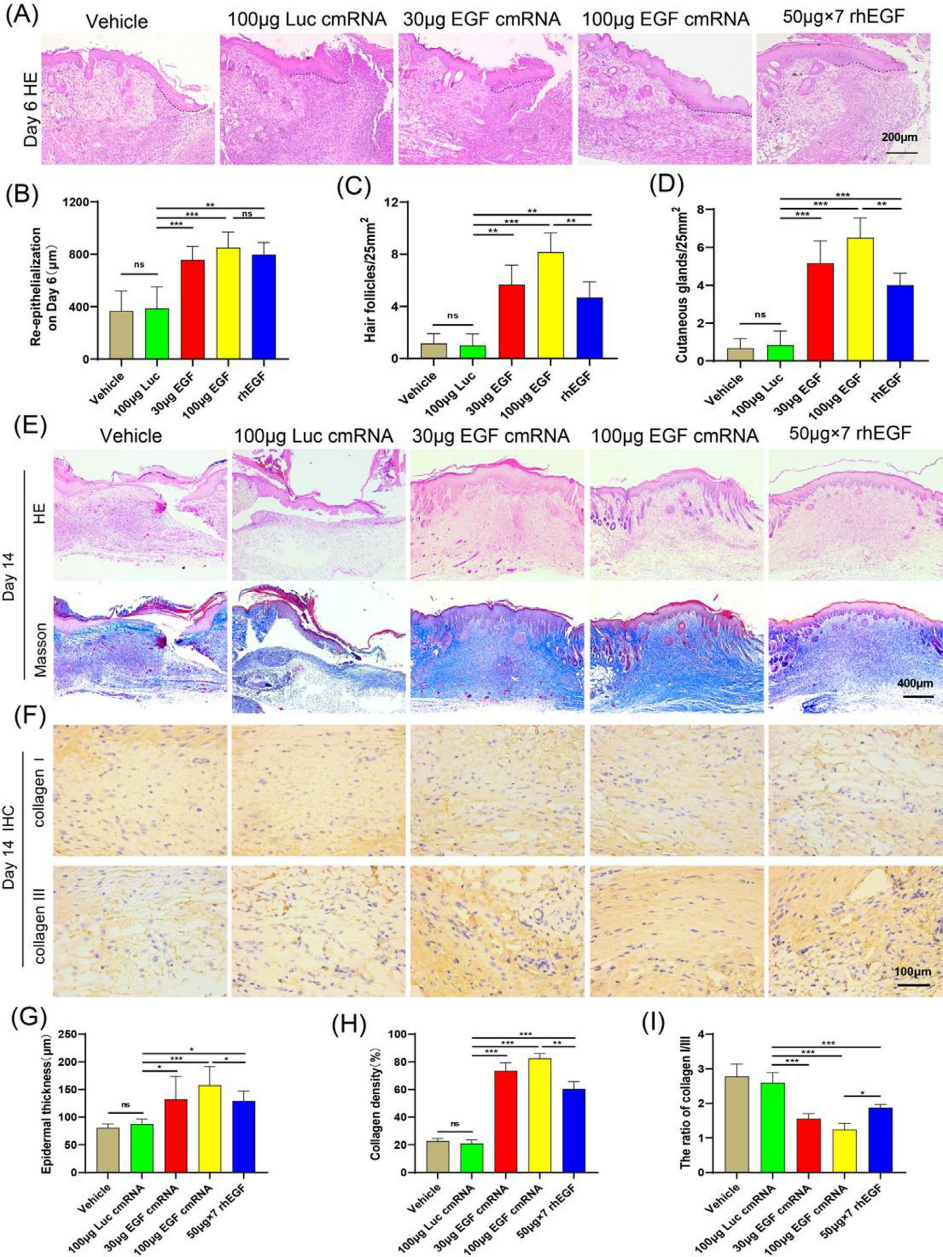
Histological analysis of wound tissues. (A) Display of representative micrographs depicting regenerated wound tissues stained with H&E on the 6th day. (B) Quantitative assessment presenting the re‐epithelialisation length (μm) on the 6th day. (C, D, G) Quantitative evaluations of cutaneous glands, hair follicles, and epidermal thickness through H&E staining. (E, F) Illustration of representative images depicting regenerated wound tissues stained with H&E, Masson's trichrome, and Collagen I and III immunohistochemistry. (H) Quantification of collagen density percent observed in Masson's trichrome staining. (I) Quantitative analysis illustrating the ratio of Collagen I/III. Data presented as mean ± SD (*n* = 5). Statistical significance is indicated as (*p < 0.05, **p < 0.01, and ***p < 0.001).

On the 6th and 14th days post‐surgery, we conducted histological staining to further evaluate the impact of EGF‐encoding cmRNA on wound tissue regeneration. On the 6th day post‐surgery, we performed H&E staining on the tissue surrounding the wound. We observed an increase in epidermal thickness across all groups, indicating active tissue regeneration post‐injury (Figure 6A). Nonetheless, the increase in epidermal thickness was more pronounced in all EGF treatment groups (30 μg, 100 μg, rhEGF). We measured the length of re‐epithelialisation at the wound edge, and the results showed that the re‐epithelialisation length in all EGF treatment groups was significantly higher than in the control groups (vehicle and 100ug Luc), but there was no statistical difference among the EGF treatment groups (Figure 6B).

On the 14th day post‐surgery, we performed H&E, Masson's trichrome, and Collagen I and III immunohistochemistry. Histological analysis of skin samples stained with H&E revealed that lesions in the vehicle and Luc groups had not fully re‐epithelialised, and there was a prominent inflammatory response. In contrast, the defects in the various EGF treatment groups disappeared and were covered by new, complete epithelial cells (Figure 6E). There was also a significant increase in the number of newly formed skin appendages, such as hair follicles and cutaneous glands, and the epidermis showed a similar thickening trend (Figure 6C,D,G). Notably, the number of skin appendages (cutaneous glands and hair follicles) in the 100 μg EGF cmRNA group was significantly higher than in the rhEGF group (Figure 6C,D).

Results from the Masson's trichrome stain indicated that a large deposition of collagen signified the recovery and maturation of the damaged tissue. The collagen fibres in the Vehicle and Luc groups were loosely and irregularly arranged, while those in the 30 and 100 μg EGF‐encoding cmRNA groups were densely and orderly aligned. Although the arrangement of collagen fibres in the rhEGF group was superior to the control groups, it was not as good as the EGF‐encoding cmRNA groups (Figure 6E). From the quantified collagen deposition results, the 100 μg EGF‐encoding cmRNA group had the highest collagen deposition rate, significantly higher than the rhEGF group (Figure 6H). In conclusion, the healed wound tissue is closer to the normal skin tissue structure after EGF‐encoding cmRNA treatment (Figure S3).

Immunohistochemical staining results for Types I and III collagens in each group revealed that the wound tissues in the Vehicle and Luc groups had more Type I collagen and relatively lower amounts of Type III collagen. In the EGF treatment groups, wound tissues had higher levels of Type III collagen and comparatively lower levels of Type I collagen (Figure 6F), which is consistent with the previously reported. Type III collagen is a newly synthesised collagen and plays a vital role in wound repair [8]. The quantitative analysis led us to the following conclusions: the ratio of Collagen I/III in the wound surface of the EGF treatment groups was significantly lower than the control groups, and the 100 μg EGF‐encoding cmRNA group was significantly lower than the rhEGF group (Figure 6I).

Furthermore, we performed H&E staining on major organs like the heart, liver, spleen, lungs, and kidneys to assess its biosafety in vivo. The results showed no significant differences among the groups (Figure S4).

### 3.6 The Effect of EGF‐Encoding cmRNA on the Expression of MEK/ERK and Ki67 In Vitro and In Vivo

**FIGURE 7:**
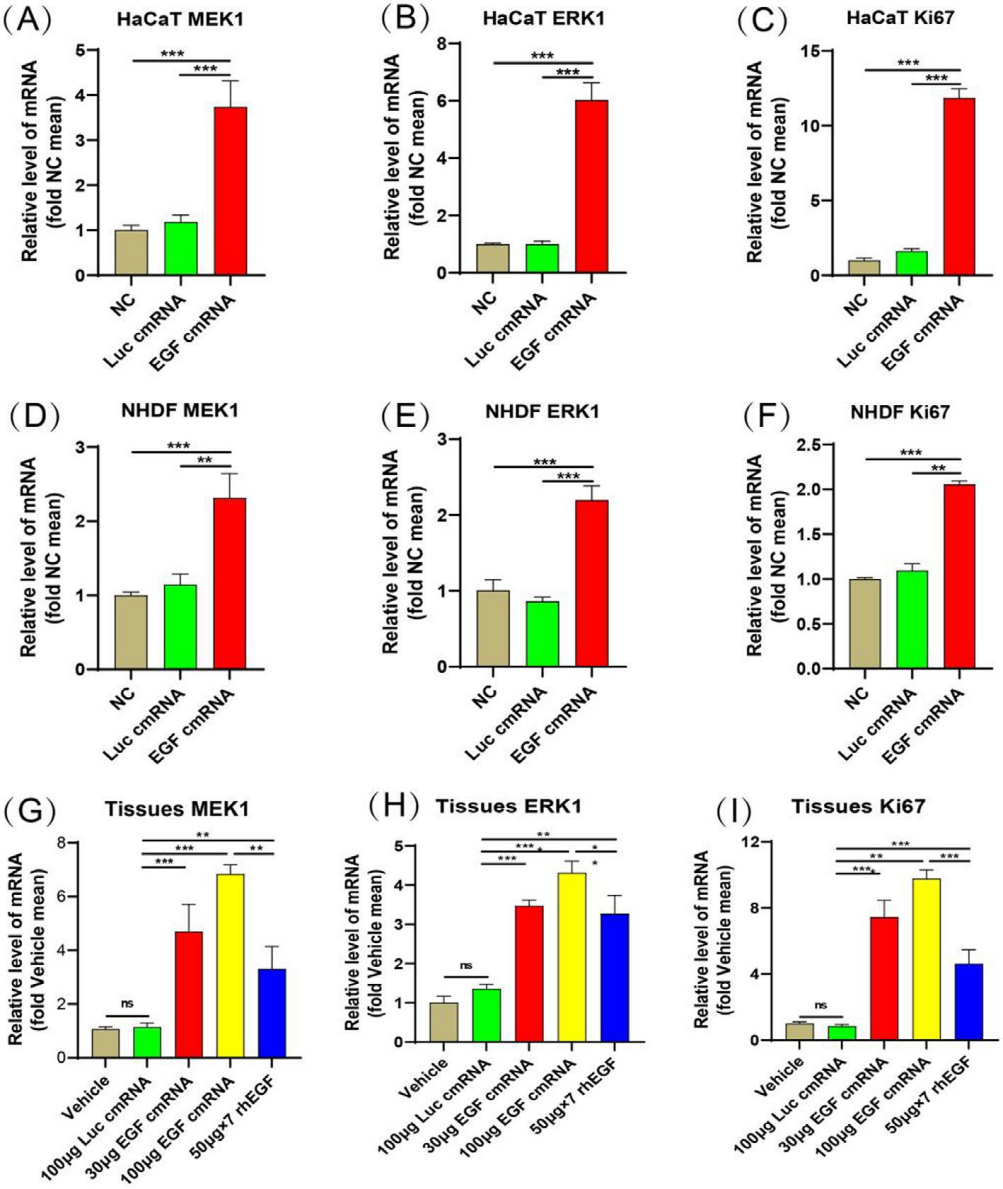
The effect of EGF‐encoding cmRNA on the expression of MEK/ERK and Ki67 in vitro and vivo. (A–C) qRT‐PCR analysis depicting the expression levels of MEK/ERK and Ki67 genes in HaCaT cells; (D–F) qRT‐PCR analysis illustrating the expression levels of MEK/ERK and Ki67 genes in NHDF cells; (G–I) qRT‐PCR assessment of MEK/ERK and Ki67 gene expression in wound tissues on the 6th day post‐treatment. Data presented as mean ± SD (*n* = 5). Statistical significance is indicated as (**p < 0.01, ***p < 0.001).

Finally, we further investigated the impact of EGF‐encoding cmRNA on both the MEK/ERK signalling pathway and Ki67 mRNA levels in vitro and in vivo. In HaCaT and NHDF cells, when compared to the NC and Luc cmRNA groups, there was a marked increase in the expression of MEK/ERK and Ki67 post‐transfection with EGF‐encoding cmRNA (Figure 7A–F). In vivo, when examining wound tissues on Day 6, results demonstrated that, relative to the Vehicle and Luc cmRNA groups, there was a pronounced augmentation in the expression of both MEK/ERK and Ki67 in the EGF‐treated groups (Figure 7G–I). Furthermore, the expression levels in the 100 μg EGF‐encoding cmRNA group significantly surpassed those in the human rhEGF group. These findings suggest that EGF‐encoding cmRNA has the capacity to activate the MEK/ERK signalling pathway, subsequently bolstering cellular capacities for proliferation and migration. Moreover, the efficacy of cmRNA appears to surpass that of the recombinant protein.

## 4 Discussion

Conventional clinical use of EGF has predominantly relied on recombinant protein technology, encountering challenges related to constrained diffusion distance, abbreviated half‐life, vulnerability to hydrolysis, and susceptibility to enzymatic degradation. The effectiveness of recombinant proteins often hinges on dosage and duration, necessitating high and recurrent doses, leading to potential adverse effects and elevated treatment expenses [36].

The recent attention towards mRNA therapies, particularly after the successful development of mRNA‐based vaccines for COVID‐19, has ignited significant interest in this innovative approach [37, 38]. Chemically modified mRNA (cmRNA), when locally administered, infiltrates the cells within the wound tissue, facilitating continuous transcription and translation of growth factors with refined bioactive structures compared to recombinant proteins. Subsequently, these factors are secreted, establishing comprehensive contact with local wound tissue cells, potentially achieving the intended therapeutic effect with reduced dosages [22, 39].

In the intricate journey of wound healing, the intrinsic ability of cells to both proliferate and migrate efficiently to the wound site stands as a cornerstone for successful tissue regeneration and repair [3, 4]. This cellular dynamism ensures that the wound is rapidly closed, minimising the risk of infections and other complications. The HaCaT and NHDF cells, representing epidermal and dermal cell types respectively, play pivotal roles in this regenerative process [40, 41], and are all regulated by EGF [42, 43]. Our study validated that EGF‐encoding cmRNA, when introduced in vitro, led to effective EGF expression in HaCaT and NHDF cells. Moreover, our findings, which highlight a significant enhancement in the proliferation and migration capacities of these cells post‐transfection with EGF cmRNA, underscore the potential therapeutic implications of this approach.

Effective uptake and utilisation of growth factors at the wound site is a crucial aspect of rapid wound healing [44, 45]. The application of recombinant proteins at the wound site is limited by their limited diffusion distance, short half‐life, and susceptibility to hydrolysis at the wound site. Their therapeutic efficacy is dose‐ and time‐dependent, often limiting their therapeutic effects, requiring long‐term high doses and/or repeated administration. Such supraphysiological doses may lead to severe side effects and increase treatment costs [36]. In contrast, cmRNA, when locally injected, enters the cells of the wound tissue and continuously transcribes and translates into growth factors with more precise bioactive structures than recombinant proteins, through paracrine action, these proteins achieve more extensive contact with surrounding wound tissues, resulting in a more potent therapeutic effect [22, 39]. Our in vivo protein expression kinetics data indicate that cmRNA can be expressed locally for a long time, and through immunohistochemistry, we found that the expressed proteins are distributed throughout the entire layer of the skin, an effect that is unachievable with the application of recombinant proteins. This prolonged expression may reduce the need for repeated treatments. In our full‐thickness skin defect model in mice, although the groups receiving two injections of 30 and 100 μg EGF‐encoding cmRNA displayed comparable re‐epithelialisation and healing rates compared to the 50 μg rhEGF administered seven times, the cmRNA‐ administered groups exhibited enhanced repair outcomes. This was evidenced by the lower ratio of Collagen I/III, increased skin appendage count, improved collagen arrangement and deposition. However, although the 100 μg group performed slightly better than the 30 μg group, no significant difference was observed. This might be due to the high concentration of EGF reducing the number and activity of EGFR, thereby diminishing the promoting effect of EGF [8]. Therefore, a further exploration of the optimal concentration is necessary in the future.

Finally, we further explored the potential molecular mechanisms underlying our findings. Previous research indicates that the MEK/ERK pathway is a fundamental cascade activated upon EGF receptor binding, orchestrating crucial biological responses like proliferation, migration, and differentiation, thus aiding in wound healing [42, 46, 47]. Concurrently, Ki67 stands as a well‐recognised protein marker indicative of cellular proliferation. Leveraging this foundational knowledge, we further investigated the influence of EGF‐encoding cmRNA on both the MEK/ERK signalling pathway and Ki67 mRNA levels, both in vitro and in vivo. Remarkably, HaCaT and NHDF cells treated with EGF‐encoding cmRNA exhibited a significant surge in MEK/ERK and Ki67 mRNA expression levels. Moreover, on the 6th day post‐wounding, the capacity of EGF‐encoding cmRNA to activate the MEK/ERK signalling pathway outpaced that of rhEGF. Maybe this is the underlying mechanism why the EGF cmRNA‐treated groups works better than the EGF recombinant protein in the skin defect experiment.

Despite the promising outcomes of our study, it is important to acknowledge several limitations. First, the localised administration of cmRNA necessitates injections into the skin tissue, which might pose discomfort to patients, even if the frequency of administration is significantly reduced. Future developments in delivery methods, such as utilising hydrogels, may alleviate this challenge. Second, our observations were confined to young, healthy mice with induced skin defects. While this scenario might be representative of specific wound patient cohorts, its applicability to diverse populations, particularly those with conditions like diabetic foot ulcers, warrants further exploration. Additionally, our exploration was confined to primary potential mechanisms, necessitating deeper investigations into the precise molecular pathways involved. Furthermore, since wound healing involves multiple stages, each regulated by different growth factors, relying on a single growth factor to address all these stages may not be sufficient to produce clinically meaningful results. Future research should focus on optimising combinations of various growth factors. Finally, due to the significant differences in wound healing mechanisms and skin structure between rodents and humans, the results of this study cannot be directly extrapolated to human clinical settings. Before considering the clinical application of our strategy, thorough studies in larger animal models, such as pigs, are essential to validate its efficacy, safety, and pharmacodynamics. These models more closely mimic human skin physiology and wound healing processes, allowing for a more accurate assessment of the strategy's effectiveness [8, 39].

In summary, the comprehensive data presented in this study stands as the initial evidence showcasing the superior efficacy of EGF‐encoding cmRNA in a biocompatible citrate buffered formulation over recombinant EGF protein in healing full‐thickness skin defects in mice. These findings indicate that EGF‐encoding cmRNA holds promising potential and could become an effective treatment for wound healing in the future.

## Ethics Statement

Experimental protocols involving animals were approved by the Institutional Animal Care and Use Committee of the First Affiliated Hospital of Nanchang University (approval no. CDYFY‐IACUC‐202311QR055). The study involving human skin samples was approved by the ethics committee of the First Affiliated Hospital of Nanchang University (ethical permission number: (2023) CDYFYYLK(10‐005)).

## Conflicts of Interest

Q.S., S.W., Y.L., C.F., J.L., F.Y. and G.W. are inventors on a provisional patent application. F.Y. and G.W. are founders and shareholders of Geneheal Pharmacy Ltd. The other authors declared no potential conflicts of interest.

## Supporting information


**Data S1** Supporting Information.

## Data Availability

All data and materials supporting the findings of this manuscript are presented in the paper and/or the Supporting Information. Additional data are available from the corresponding authors upon reasonable request.
