# Supplementary material for: Enhanced Skin Wound Healing Through Chemically Modified Messenger RNA Encoding Epidermal Growth Factor (EGF)
Source: Int Wound J. 2025 May 4;22(5):e70143. doi: 10.1111/iwj.70143 (PMC12050261; doi:10.1111/iwj.70143)

**Supplementary Tables and Figures**

Supplementary Table 1. Tail PCR primers.

| Primers | Sequence (5'-3') |
| --- | --- |
| F1 | GAATTGACGCGTATTGGGATTAATACG |
| R1-T120 | TTTTTTTTTTTTTTTTTTTTTTTTTTTTTTTTTTTTTTTTTTTTTTTTTTTTTTTTTTTTTTTTTTTTTTTTTTTTTTTTTTTTTTTTTTTTTTTTTTTTTTTTTTTTTTTTTTTTTTTTGCCGCCCACTCAGATTTATTCAAAGAC |

Supplementary Table 2. Open reading frame sequence for construction of human EGF

cmRNA

| Human  EGF | ATGCTGCTGACCCTGATCATCCTGCTGCCCGTGGTGAGCAAGTTCAGCTTCGTGAGCCTGAGCGCCAACAGCGACAGCGAGTGCCCCCTGAGCCACGACGGCTACTGCCTGCACGACGGCGTGTGCATGTACATCGAGGCCCTGGACAAGTACGCCTGCAACTGCGTGGTGGGCTACATCGGCGAGAGATGCCAGTACAGAGACCTGAAGTGGTGGGAGCTGAGATGA |
| --- | --- |

Supplementary Table 3. 5′UTR and 3′UTR sequence.

| 5′UTR | GGGGAAATAAGAGAGAAAAGAAGAGTAAGAAGAAATATAAGACCCCGGCGCCGCCACC |
| --- | --- |
| 3′UTR | TAATAGGCTGGAGCCTCGGTGGCCTAGCTTCTTGCCCCTTGGGCCTCCCCCCAGCCCCTCCTCCCCTTCCTGCACCCGTACCCCCGTGGTCTTTGAATAAAGTCTGAGTGGGCGGC |

Supplementary Table 4. Human primers used in qRT-PCR analysis.

| Primers | Sequence (5'-3') | Product size(bp) |
| --- | --- | --- |
| MEK-F | TGAAGCTGGAGAGGACCAAC | 105 |
| MEK-R | CTCCCACCTTCTGCTTCTGG |
| ERK-F | TCAGACTCCAAAGCCCTTGAC | 143 |
| ERK-R | GACTGGCCCACCTCATCC |
| Ki67-F | GGATCGTCCCAGTGGAAGAG | 128 |
| Ki67-R | CAAACAAGCAGGTGCTGAGG |
| GAPDH-F | AATGGGCAGCCGTTAGGAAA | 114 |
| GAPDH-R | GCCCAATACGACCAAATCAGAG |

Supplementary Table 5. Mouse primers used in qRT-PCR analysis.

| Primers | Sequence (5'-3') | Product size(bp) |
| --- | --- | --- |
| MEK-F | GATGAAGCTGGAGAGGACCAA | 123 |
| MEK-R | CTTGAAGACCACTCCACCGT |
| ERK-F | CCCAAGTGATGAGCCCATTG | 113 |
| ERK-R | CTTACACCATCTCTCCCTTGCT |
| Ki67-F | ACCATCATTGACCGCTCCTT | 109 |
| Ki67-R | TTGACCTTCCCCATCAGGGT |
| GAPDH-F | CCAATGTGTCCGTCGTGGAT | 98 |
| GAPDH-R | TGCCTGCTTCACCACCTTCT |

Supplementary Figure 1. Depiction of a schematic illustrating the application of EGF-encoding cmRNA for skin wound healing.


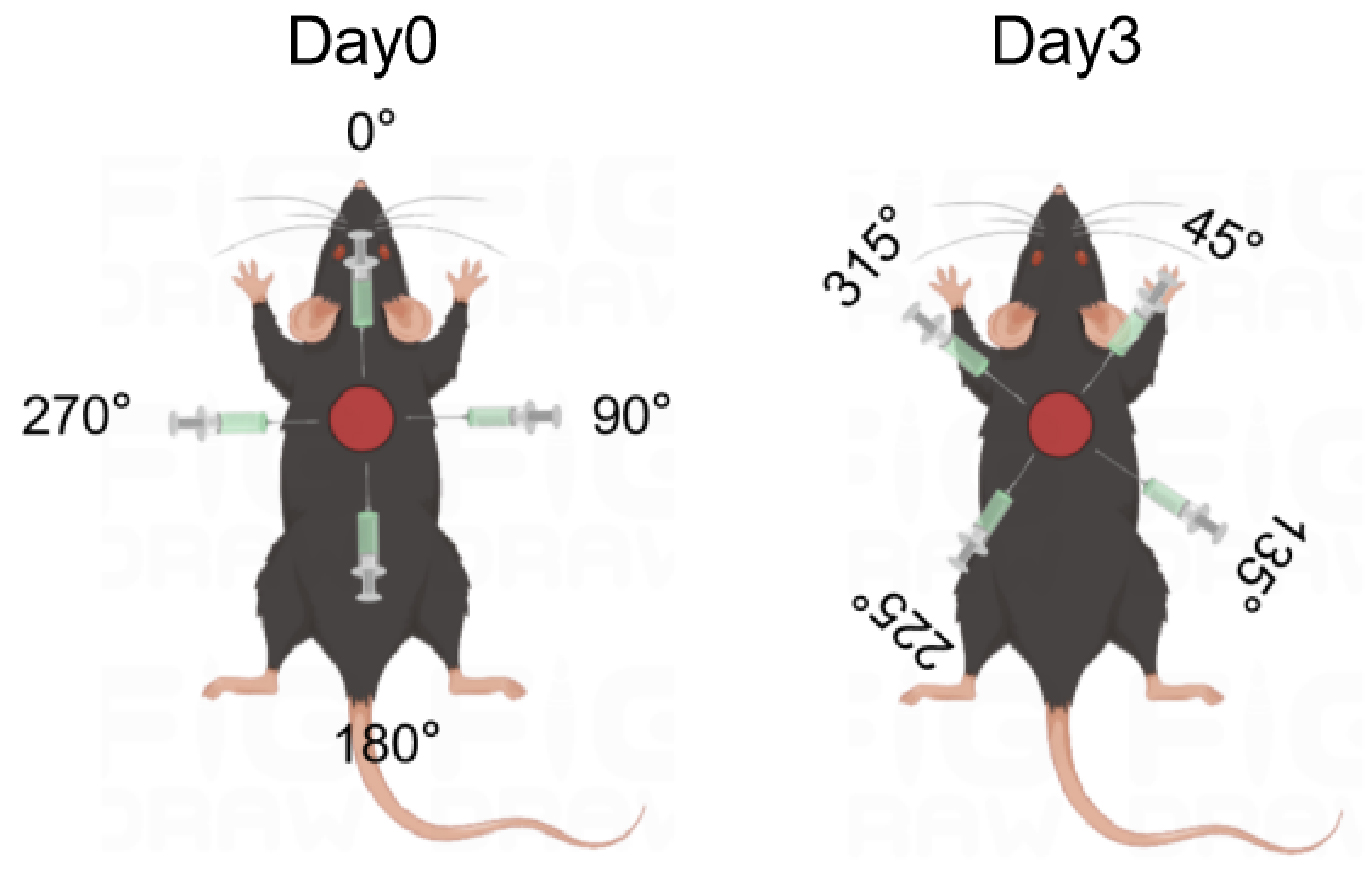


Supplementary Figure 2. Flow chart of the animal experimental design.


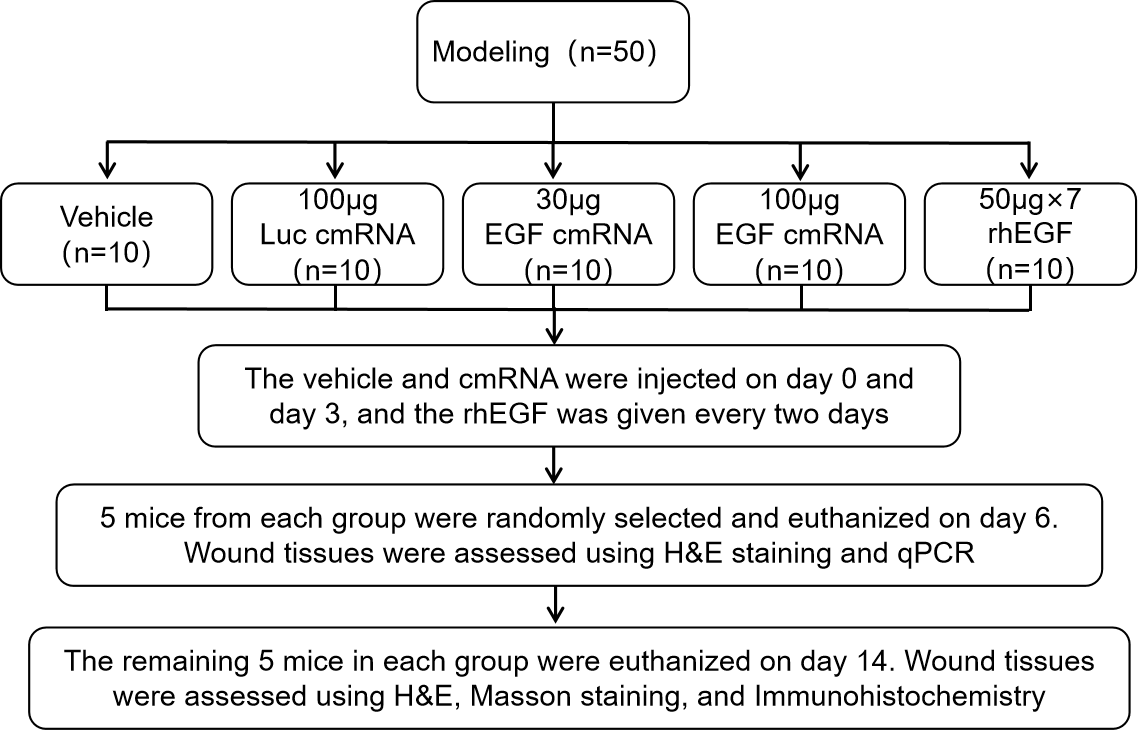


Supplementary Figure 3. Depiction of representative images displaying normal skin stained with H&E and Masson’s trichrome.


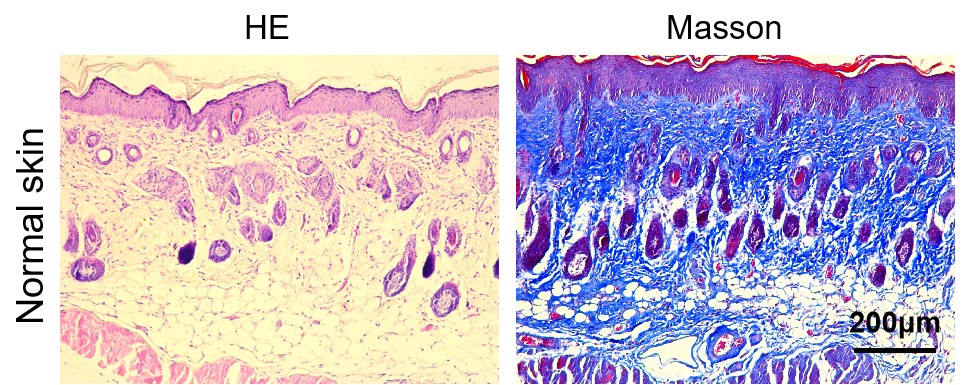


Supplementary Figure 4. Depiction of representative images displaying major organs, including the heart, liver, spleen, lung, and kidney, stained with H&E.


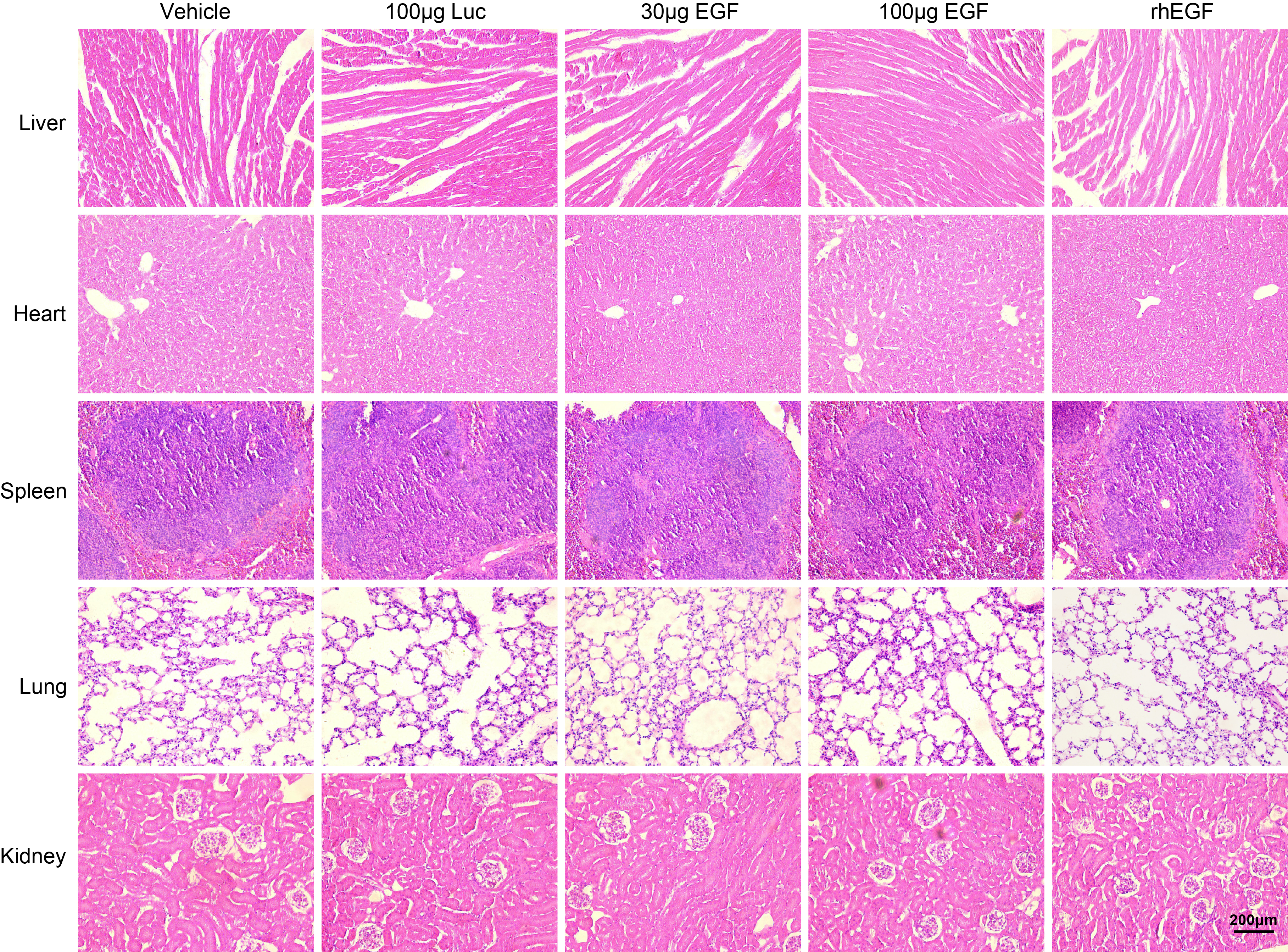

Supplement: Supplementary file 1 — Data S1 Supporting Information. [file IWJ-22-e70143-s001.doc]
